# Promoting Identity Development, Multicultural Attitudes, and Civic Engagement Through Ethnic Studies: Evidence From a Natural Experiment

**DOI:** 10.1111/cdev.14219

**Published:** 2025-01-20

**Authors:** Sarah Gillespie, Mirinda M. Morency, Elizabeth Fajemirokun, Gail M. Ferguson

**Affiliations:** ^1^ Institute of Child Development University of Minnesota Twin Cities Minneapolis Minnesota USA

**Keywords:** ethnic‐racial identity, ethnic studies, global competence

## Abstract

This study used a natural experiment design to examine the impact of ethnic studies courses on students' ethnic‐racial identity (ERI) development, multicultural attitudes, and civic engagement during the 2021–2022 school year in Minneapolis, MN (*N* = 535; 33.5% White, 29.5% Black, 21.1% Latine, 10.7% multi‐racial; 44.7% female, 7.1% non‐binary). Compared to students who were quasi‐randomly assigned to a control class, 9th graders taking an ethnic studies class (treatment group) engaged in significantly more midpoint ERI exploration (*β* = 0.12), resulting in stronger endpoint ERI resolution (*β* = 0.48–0.57). Increased exploration mediated more favorable attitudes toward multiculturalism (indirect effect = 0.05) and more frequent civic engagement activities (indirect effect = 0.02). Results have implications for policy efforts to expand ethnic studies.

Globalization and demographic changes have diversified the societies in which many adolescents are developing their worldviews, sense of identity, and attitudes about individuals from different backgrounds. The associated opportunities and challenges raise questions, which psychologists are well‐equipped to answer, about what it means for individuals and communities to thrive in the superdiverse societies of the 21st century (Vertovec 2019). In these contexts, a deeper understanding of one's own ethnic‐racial identity (ERI; Umaña‐Taylor 2023), viewing diversity as an asset rather than a threat (Chen et al. 2016), and shaping the direction of society through civic and political participation (Jang, Schwarzenthal, and Juang 2023; López et al. 2022) are all salient skills for both individual and collective well‐being. Ethnic studies (ES) classes, which encourage ERI development and increase sociopolitical awareness through a critical analysis of racism and social movements throughout history and in the present day (Sleeter and Zavala 2020), are one promising avenue for promoting youths' positive development in diverse communities and for disrupting the transmission of racist ideologies and systems across generations (Ferguson et al. 2022; Leslie et al. 2020). The present study capitalized on a natural experiment to evaluate the impact of a new ES graduation requirement on high schoolers' ERI exploration and resolution, attitudes toward multiculturalism, and civic engagement.

## 1 Development of ERI in Schools

Identity development intensifies during adolescence as young people look inwards, to interrogate their values and beliefs, and outwards, to understand their role in society (Erikson 1968; Umaña‐Taylor et al. 2014). In societies stratified by racism and xenophobia like the United States, developing one's ERI supports youth with marginalized ethnic‐racial identities (hereafter: “students of color,” reflecting preferred language in the study sample) by conferring pride, belonging, and collective efficacy within one's ethnic‐racial group (Umaña‐Taylor et al. 2014, 2018). Taking steps to learn about one's ethnic or racial heritage (ERI exploration) and resolving feelings about belonging to one's ethnic‐racial group (ERI resolution) are associated with resilience in the face of historical trauma and interpersonal and structural racism (Neblett, Rivas‐Drake, and Umaña‐Taylor 2012; Rivas‐Drake et al. 2022). For White adolescents, development of their ERI also entails understanding racial privilege and the ability to define a positive, moral White identity through self‐exploration and antiracist action (Helms 2019; Moffitt, Rogers, and Dastrup 2022).

During adolescence, schools and peer groups become an increasingly important environment for ERI exploration and socialization (Rivas‐Drake et al. 2017; Stein et al. 2018). ES classes—which include an explicit focus on the histories and cultures of racial groups and a critical analysis of racism—have been shown to increase ERI development, awareness of racism, and academic achievement for students in elementary school through college (see review by Sleeter and Zavala 2020). Studies evaluating universal ES programs or ERI‐focused interventions with robust research designs point to the benefits of encouraging ERI exploration for students' psychological well‐being, academic achievement, and school engagement (Bonilla, Dee, and Penner 2021; Gillespie et al. 2024; Umaña‐Taylor et al. 2018). For example, an ES evaluation in California used a natural experiment to show that ES enrollment predicted higher attendance, GPA, graduation rates, and matriculation to college among students at elevated risk for poor academic outcomes (Bonilla, Dee, and Penner 2021). Another natural experiment in the Midwest found that taking an ES class relative to a control class was associated with ERI exploration and resolution development, well‐being, and academic outcomes for both students of color and White students (Gillespie et al. 2024). Finally, a randomized controlled trial (RCT) of the Identity Project, an 8 session ERI‐focused intervention delivered in classrooms, found that increased ERI exploration and resolution mediated the benefits of the program for students of color and White students alike, which included improved outgroup orientation, grades, academic engagement, self‐esteem, and lower depressive symptoms (Umaña‐Taylor et al. 2018). That noted, change in ERI resolution occurred more rapidly for students of color and for those receiving more ERI socialization at home (Sladek et al. 2021), pointing to the need for analyses that account for students' positionality.

Nevertheless, strong political opposition to discussions of identity and racism in U.S. schools has been stoked by fears that these classes are divisive and increase racial animosity between students. Many existing studies have examined individual outcomes, such as academic achievement or well‐being, while fewer have analyzed the possible impacts at the school and community level via changing attitudes toward diversity or increased civic engagement. As states consider expansion or bans on these courses, there is a particular need for more research on the effects of universal ES curricula delivered in classrooms comprised of students from diverse ethnic‐racial backgrounds.

## 2 Global Competence and Adolescent Adaptation in Multicultural Societies

In diversifying, globally connected societies, scholars have defined *global competence* as the ability to critically examine societal issues, engage with the perspectives of others, interact effectively across cultural difference, and work collectively toward goals that benefit society (Deardorff 2006; Jang, Schwarzenthal, and Juang 2023). Proactive, positive attitudes toward multiculturalism and civic engagement are two manifestations of global competence among adolescents. ERI exploration and resolution could support each of these domains (Rivas‐Drake et al. 2022).

### 2.1 Multicultural Attitudes and ERI

Individuals vary in their attitudes toward globalization and multiculturalism, which has implications for individuals' own well‐being in diverse societies and the resilience of their communities. Favorable *multicultural attitudes* involve a proactive, eager stance toward learning about other cultures, recognizing and valuing diversity in society, and engaging with multicultural settings (Chen et al. 2016). Multicultural attitudes have been linked to better psychological adaptation, higher sociocultural competence, and more positive attitudes toward other groups among college students and adults in Hong Kong, Canada, mainland China, and the United States (Chen et al. 2016; Ferguson et al. 2022), making them an indicator of adaptation within multicultural societies. A recent meta‐analysis that included primarily adult research also linked favorable multicultural attitudes to lower discrimination, higher quality relationships with outgroup members, and greater support for diversity‐related policies (Leslie et al. 2020). These attitudes, positive interactions, and the policy and social changes they engender can increase social cohesion within a society. More distally, broad changes in individual attitudes could lead to greater support for policy changes and social movements that are necessary to dismantle structural racism (McGee 2021; Metzl 2019).

The concepts of ERI exploration and multicultural attitudes share psychological and behavioral signatures, including curiosity, openness, and strong motivation to engage with new people or information. ES classes and ERI‐focused programs position exploration of one's own identity and the cultures and histories of outgroup members as explicit goals and scaffold this process via assignments, strategies, and discussions (Umaña‐Taylor, Sladek, and Safa 2024). Conveying appreciation for individuals' identities in the classroom may also support self‐esteem and a sense of high public regard, the belief that others view one's ethnic‐racial group positively, that have been linked to favorable outgroup attitudes (Wantchekon et al. 2022, 2023).

ERI exploration facilitated by close student–teacher relationships and effective strategies may also help students overcome some of the challenges linked to the ERI development process, including increased vulnerability to discrimination or outgroup avoidance as exploration increases for students of color (Yip et al. 2019) or strong negative emotions when confronting privilege among White individuals (DiAngelo 2016). Discrimination experiences can trigger ERI exploration (Cheon and Yip 2019), but may also discourage openness to outgroup members. By contrast, ES classes offer invitations to explore positive counternarratives about one's own and other groups, which could encourage empathy and allyship (Fish et al. 2021; Rivas‐Drake et al. 2022). Among college students of color, ERI exploration, but not resolution, predicted a greater “ally identity” toward other groups, including solidarity and sociopolitical actions (Fish et al. 2021). For White students, sustained engagement in exploring racism and White ethnic‐racial identities is inherent to advanced stages of White racial identity development (Helms 2019; Satterthwaite‐Freiman and Umaña‐Taylor, 2023). The current study tests whether ES classes, by explicitly scaffolding ERI exploration and learning about other cultures, equip adolescents with skills for living in multicultural societies.

### 2.2 Civic Engagement and ERI

Adolescents have access to many civic engagement activities, such as volunteering, protesting, signing petitions, joining organizations, and communicating about civic issues. They are also preparing for voting by exploring political identities and systems. Civic engagement offers feelings of individual and collective efficacy, conveying a sense that their voice and actions can shape the direction of society (Maker Castro, Wray‐Lake, and Cohen 2022). Higher participation in civic engagement during adolescence and emerging adulthood is prospectively linked with higher income and education and better mental health in adulthood (Ballard, Hoyt, and Pachucki 2019). Civic engagement during adolescence is, therefore, an indicator of positive development and future well‐being in diverse democracies.

ES classes could increase students' civic engagement by fostering cross‐racial interactions, encouraging exploration of their social identities, and increasing awareness of historical movements for social justice. ERI helps students to engage effectively in multicultural interactions, which predict higher civic engagement among high school students (Tarman and Kilinc 2022) and enable youth to build cross‐racial coalitions for civic action (Rivas‐Drake et al. 2022). ERI exploration may also motivate youth of color to engage in civic life through cultivating a sense of shared fate, belonging, responsibility, and solidarity (Fish et al. 2021). White students' awareness of Whiteness and sense of being in the racial majority also predicts more positive attitudes about civic engagement (Conrad, Lo, and Kisa 2022; Satterthwaite and Umaña‐Taylor 2023). Finally, ES classes provide examples of successful civic action (Haduong et al. 2023), which can help adolescents resist demoralizing or negative narratives that discourage their civic participation (Hope, Pender, and Riddick 2019). The current study explores the role of ES classes and ERI exploration and resolution in promoting adolescents' civic engagement.

## 3 Current Study

We used a natural experiment design to strengthen causal inferences about the impact of ES courses on the ERI exploration and resolution, multicultural attitudes, and civic engagement of students of color, including Black, Latine, Indigenous, Asian, and multiracial students, and White high school students. Following quasi‐random assignment to take an ES class (treatment) or a standard social studies course (control; U.S. Government or Human Geography) after the passage of a new ES graduation requirement, we attributed differences in attitudes and behaviors to the course itself rather than pre‐existing characteristics of the students. These circumstances provide important insights into the feasibility, scalability, and impact of ES classes when implemented in these types of settings. Existing teachers operating in ethnic‐racially diverse classrooms developed an ES curriculum based on four targets within the district's framework: explicit exploration of identity, structural analysis of racism, counternarratives of communities of color, and interdisciplinary learning that leads to action. A steering committee comprised of educators, equity specialists, and ES faculty at a local university developed the framework and supported teachers' work throughout the school year. https://doi.org/10.17605/OSF.IO/BDKTY.

We analyzed several pre‐registered, confirmatory hypotheses (deidentified link) using longitudinal structural equation modeling (SEM) of survey data collected at the midpoint and endpoint of the semester‐long course. Aim 1 tested the effects of ES classes on ERI exploration and resolution. Based on prior literature demonstrating that ES classes catalyze ERI development (Sleeter and Zavala 2020; Thomas, Davidson, and McAdoo 2008; Wiggan and Watson‐Vandiver 2019), we hypothesized that ES students would have higher ERI resolution by the end of the semester, mediated by higher ERI exploration at the midpoint (Hypothesis 1; H1). We also hypothesized that the impact of the ES class on ERI exploration and resolution would be stronger for White students than students of color (H2), since the course would be more novel to White students who typically experience lower rates of racial socialization (Loyd and Gaither 2018). While the Identity Project found that ERI resolution changed more rapidly among students of color than White students during an 8‐week intervention (Sladek et al. 2021), the extended duration of this ES class (20 weeks) could allow time for White students to overcome any initial lag in effects.

Aim 2 tested the hypothesis that increases in ERI—in particular, ERI exploration—would mediate course‐related improvements in the global competence markers of multicultural attitudes (H3) and civic engagement (H4) for ES relative to control students (Rivas‐Drake et al. 2022). The district's ES framework includes exploration of one's own ERI and the history of racism faced by different groups as key foundations for critical action. Consistent with this theory of change, we predicted that ERI exploration, which involves active cultural engagement and high motivation, would be a stronger mediator than resolution for both outcomes. ERI exploration, but not resolution, has been linked to allyship and sociopolitical behavior among students of color (Fish et al. 2021), and a lifelong commitment to self‐exploration and antiracist action marks more advanced stages of White racial identity development (Helms 2019).

## 4 Method

### 4.1 Study Design

**FIGURE 1:**
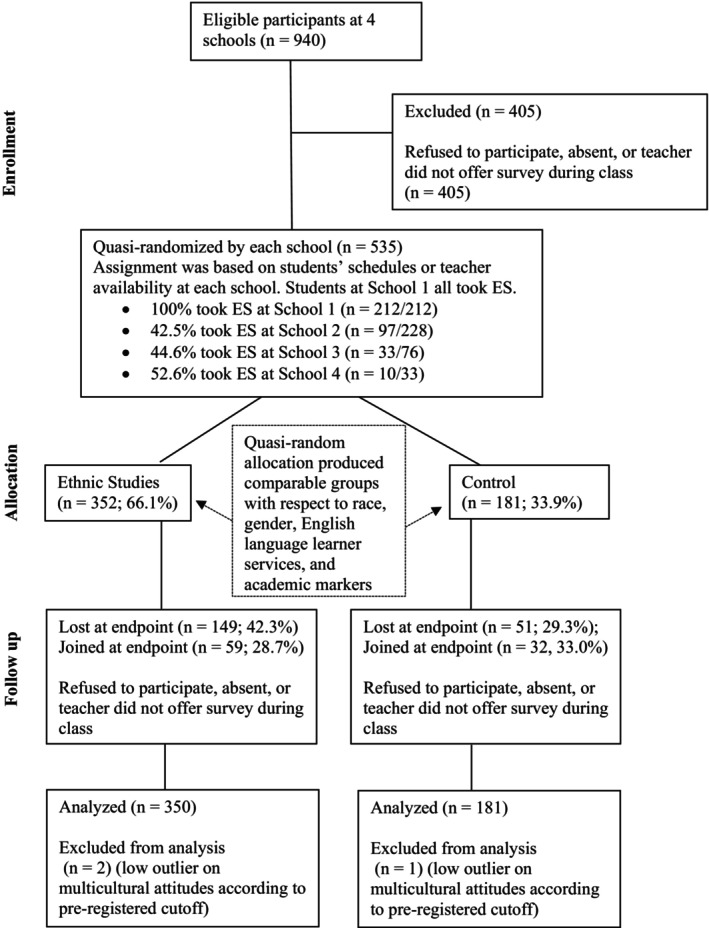
Diagram of the quasi‐randomization procedure and analytic sample.

Study procedures were approved by the University of Minnesota's IRB and the school district's research department. All school principals in the district were contacted about the study, and four opted into participation for the 2021–2022 academic year. At these schools, the ES class was included in the 9th grade sequence (either fall or spring semester), so the study team invited all 9th grade teachers offering either ES or control classes to participate; some teachers taught both subjects, while others taught only one. Students were quasi‐randomly assigned to either the ES or control class by procedures within their individual school, such as their overall course schedules or teacher availability (Figure 1). At all but one school, approximately half of students took ES, providing a within‐school control group; at School 1, all students took ES. We obtained passive consent by notifying 9th‐grade parents of the program evaluation project via school letters in English, Somali, Hmong, and Spanish. All students were read instructions by their teacher and informed of their rights as study participants before proving active assent and completing online surveys at the midpoint (November) and endpoint (January–February) during their ES (ES = 1) or control class (control = 0) using cell phones or school‐issued laptops.

### 4.2 Participants

The final sample included 535 9th‐grade students from four high schools in Minneapolis, MN. The sample was diverse and representative of district demographics, with 33.5% identifying as non‐Latine White and 66.5% students of color identifying as 29.5% Black or African American, 21.1% Latine, 10.7% bi‐ or multi‐racial, 2.8% Asian, 2.2% Native American, and 0.2% Pacific Islander. We grouped participants into “Student of Color” and non‐Latine “White” based on their self‐reported demographics. Students self‐identified as male (48.2%), female (44.7%), and non‐binary gender (7.1%). While immigrant background was not collected, 16.8% of the sample received English Language Learner services.

Participating schools predominantly served students of color, ranging from 58.6% (School 2) to 100% (School 4) of the student body. Most students across schools participated in the free or reduced lunch program, ranging from 59% (School 2) to 96% (School 4). All ES and control teachers at Schools 1 and 2 were White and taught a broad ES class that included the histories of many different ethnic‐racial groups, representing a majority of the sample (87.78%), while teachers at Schools 3 and 4 were more likely to be teachers of color and to teach courses that were specific to their own heritage or the heritage of students at that school.

### 4.3 Measures

#### 4.3.1 ERI

We measured ERI development with the Exploration and Resolution subscales of the Ethnic Identity Scale (Umaña‐Taylor, Yazedjian, and Bámaca‐Gómez 2004) at the midpoint and endpoint of the semester. Seven exploration items (e.g., “I have attended events that have helped me learn more about my ethnicity”) and four resolution items (e.g., “I am clear about what my ethnicity means to me”) were rated on a four‐point Likert scale from 1 (*Does not describe me at all*) to 4 (*Describes me very well*). We omitted one reverse‐scored item from the exploration scale following confirmatory factor analysis (CFA) of the factor loadings, factor structure, and measurement invariance (configural for exploration and scalar invariance for resolution; see Supporting Information S1), meaning six items were included as indicators of exploration and four items as indicators of resolution. In sensitivity analyses, all ten items were loaded onto a single latent variable. Cronbach's alphas were acceptable at midpoint (White *α* = 0.89; students of color *α* = 0.86) and endpoint (*α*s = 0.87 & 0.82, respectively).

#### 4.3.2 Multicultural Attitudes

We used the Multicultural Acquisition subscale of the Global Orientations measure (Chen et al. 2016) to assess attitudes toward diversity and proactive engagement with multiculturalism at the semester endpoint. Participants rated their responses to 13 items (e.g., “I am eager to make friends with people from different cultural backgrounds”) on a 7‐point Likert scale, and all items were included as indicators. A CFA established metric invariance, one‐factor structure, and strong loading of all items (Supporting Information S1). There was strong internal consistency for students of color (*α* = 0.92) and White students (*α* = 0.94).

#### 4.3.3 Civic Engagement

We measured civic engagement activities with the Critical Action subscale of the Critical Consciousness Scale (Diemer et al. 2015) at the endpoint of the semester. In addition to the nine original items focused on participation in demonstrations, organizations, or individual actions (e.g., “Joined in a protest march, political demonstration, or political meeting”), we added a tenth item to capture newer forms of online civic engagement (e.g., “Wrote or posted about a social or political issue on a social media account or blog”) and computed a mean score. Students reported their frequency of involvement in these activities over the last semester using a 4‐point Likert scale ranging from 0 (*Never did this*) to 3 (*At least once a week*), which we rescaled to reflect daily frequency (e.g., *At least once a week* = 0.14 times per day). Internal consistency was acceptable for students of color (*α* = 0.78) and White students (*α* = 0.73). Since this scale served as an index of behaviors, rather than representation of a latent variable, we did not conduct a CFA and treated it as a manifest in analyses.

### 4.4 Planned Analyses

All authors and research assistants engaged in reflective processes on their social positionality and personal developmental experiences related to study hypotheses and interpretation of findings (Jacobson and Mustafa 2019). See Supporting Information S3 for details.

All analyses were conducted in Mplus Version 8.5 using full information maximum likelihood (FIML) estimation to account for missing data. We first confirmed the factor structure and assessed measurement invariance for ERI exploration and resolution and multicultural attitudes, which were used as latent variables in SEM (see Supporting Information S1). We included dummy‐coded gender as a covariate for all variables, with males as the reference group, to reflect known gender differences in ethnic‐racial socialization and gendered experiences of privilege and oppression relevant to ERI development (Umaña‐Taylor and Hill 2020), higher multicultural attitudes among females (Chen et al. 2016), and domain‐specific gender differences in attitudes toward civic engagement (Metzger and Ferris 2013). We pre‐registered acceptable fit for SEM as *CFI* > 0.95, *RMSEA* < 0.08, *SRMR* < 0.10, and *χ* ^2^/df < 3 (Byrne 2013; Hu and Bentler 1999). The one deviation from pre‐registration was accepting model fit with *CFI* > 0.90 when all other indicators suggested excellent model fit, rather than pursuing additional modification indices that were not theoretically supported. Students were nested in classrooms within four schools, but initial examination of the intraclass correlations (ICC) showed that variance was negligible at the classroom level (exploration = 0.03; resolution = 0.02; multicultural attitudes = 0.07; civic engagement = 0.03) and below the pre‐registered threshold (ICC < 0.15) at which multilevel modeling would be needed (Irimata and Wilson 2018). Our sample of four schools is below the recommended lower limit for multilevel modeling of at least ten groups per level (Hoyle and Gottfredson 2015).

To select the final model for aims 1 and 2, we fit and compared nested models using Chi‐squared difference tests. For aim 1, we computed indirect effects using bias‐corrected 95% confidence intervals (CI) based on 1000 bootstrapped samples to test the course effect on ERI resolution, as mediated by ERI exploration (H1) and course effects on outcomes through the mediators of exploration and resolution. Using midpoint exploration allowed us to establish the temporal precedence of our key mediator for both resolution and our outcomes. A simulation study showed that a sample size of 462 is adequately powered (power = 0.80; alpha = 0.05) to detect mediation when the *α* path between the independent variable and mediator and the *b* path between the mediator and dependent variable are both small (effect size < 0.14; Fritz and Mackinnon 2007). We next used multigroup SEM to compare the equality of model parameters across students of color and White students (H2). We started with the most constrained version of the model and then released individual paths sequentially, starting with the largest differences in standardized coefficients between groups, until models were not significantly different. While there is debate about adding versus removing constraints, we expected the majority of paths to be equivalent across groups (with the ES effect on exploration and resolution as two varying paths). Therefore, our data‐driven strategy of fully constraining the model was chosen to reduce Type 1 error rates by minimizing the number of tests (Millsap and Cham 2011; Yoon and Millsap 2007).

For aim 2, we fit a constrained model where the path from midpoint ERI exploration to the outcomes was fixed to 0 and compared it to one where that path was freely estimated. This indicated whether ERI exploration operated as a mediator directly or through its influence on ERI resolution. We next tested mediation of course effects on multicultural attitudes and civic engagement using 95% CIs (H3 and H4). We checked standardized residuals for the final model to determine the local fit of the model, which did not indicate major misspecification.

## 5 Results

### 5.1 Comparison of Ethnic Studies and Control Group After Quasi‐Randomization

Table 1 reports demographics of each group, demonstrating that this process created comparable groups with respect to racial background, gender identity, and English Language Learning status (see Supporting Information S2 for additional details). Quasi‐randomization was also checked within Schools 2–4 to establish that each school's procedure for assigning ES or control classes produced comparable groups across race, English Language Learner status, gender, and middle school GPA and attendance (see Supporting Information S2). Subsequent differences between these groups can be reasonably attributed to effects of the ES class relative to the control, while acknowledging the possibility of unmeasured confounders.

**TABLE 1:** Sample demographics following quasi‐random assignment to ethnic studies or control.

	Ethnic studies		Control class		*χ* ^2^ (df); *p*
	*n*	%	*n*	%	
Racial background					11.24 (6); *p* = 0.08
1. Black or African American	119	33.8	38	21.0	
2. Latine	71	20.2	42	23.2	
3. Biracial or multiracial	34	9.7	23	12.7	
4. Asian	9	2.6	6	3.3	
5. Native American	9	2.6	3	1.7	
6. Hawaiian or other Pacific Islander	1	0.3	0	0.0	
7. White	109	31.0	69	38.1	
ELL services	61	17.3	29	16.0	0.15 (1); *p* = 0.70
Gender identity					0.67 (2); *p* = 0.71
1. Male	169	48.0	88	48.6	
2. Female	160	45.5	78	43.1	
3. Non‐binary	23	6.5	15	8.3	

### 5.2 Missing Data and Data Distributions

COVID‐19‐related impacts on student attendance on data collection days plus staffing changes resulted in a relatively high level of missingness. Students took the midpoint survey only (37.5%), endpoint only (17.4%), or both (45.1%). We report all included data by timepoint, class assignment, and racial background in Table S1. Notwithstanding, data were missing completely at random, according to Little's MCAR test (*χ* ^2^ = 579.34, *p* = 0.77). There was no systematic missingness according to levels of ERI, multicultural attitudes, or civic engagement at the midpoint, but staffing changes at School 1, where 100% of students took ES, meant that these students were more likely to be lost at the follow‐up surveys (Figure 1). Accordingly, we fit models using FIML (Dong and Peng 2013). Including variables in the model that predict missingness improves the performance of FIML, and each of these key variables operated as a predictor or covariate in our model (Graham 2003). ERI, multicultural attitudes, and civic engagement were approximately normally distributed according to skewness and kurtosis values (Hair 2009). Following the pre‐registered plan, we replaced outliers greater than 3SD from the sample mean with the mean ± 3SD value for manifest variables (6 cases for civic engagement) and excluded individuals with outliers on latent variables (3 cases for multicultural acquisition; 0 for ERI variables). Table 2 includes correlations and descriptive statistics.

**TABLE 2:** Descriptive statistics and correlations among study variables.

	1	2	3	4	5	6	7	Students of color
								*M* (SD)
1. ES class		0.12^+^	0.10	−0.05	0.03	−0.16*	−0.10	61% in ES
2. ERI exploration (midpoint)	0.13		0.61***	0.72***	0.59***	0.46***	0.31***	2.73 (0.72)
3. ERI resolution (midpoint)	0.09	0.42***		0.44***	0.64***	0.32***	0.07	3.07 (0.78)
4. ERI exploration (endpoint)	0.22*	0.64***	0.26*		0.63***	0.49***	0.34***	2.80 (0.69)
5. ERI resolution (endpoint)	0.0	0.26*	0.57*	0.37***		0.38***	0.13^+^	3.10 (0.71)
6. Multicultural attitudes (endpoint)	0.23*	0.33**	−0.13	0.38***	0.08		0.27***	5.54 (1.07)
7. Civic engagement (endpoint)	0.19^+^	0.20^+^	−0.04	0.27**	0.08	0.47***		0.40 (5.51)
White students *M* (SD)	68% in ES	2.16 (0.76)	2.60 (0.80)	2.21 (0.69)	2.61 (0.79)	5.64 (1.05)	0.47 (4.92)	

*Note:* Correlations are reported above the diagonal for students of color and below the diagonal for White students. Ethnic studies (ES) is coded 1, while the control is coded 0.

^+^ *p* < 0.1.

* *p* < 0.05.

** *p* < 0.01.

*** *p* < 0.001.

### 5.3 Aim 1: ERI Exploration and Resolution in Ethnic Studies

Consistent with H1, the ES class assignment predicted higher endpoint ERI resolution, which was mediated by higher midpoint ERI exploration (indirect effect = 0.06, 95% CI [0.01, 0.11]). Multigroup SEM tested whether paths were consistent in direction and magnitude between students of color and White students (Model 1 vs. 2 and 3 in Table 3). Contrary to H2, the paths between ES, exploration, and resolution were equivalent across racial groups.

**TABLE 3:** Model comparisons for test of mediation and multigroup equivalence.

Model comparison	Δ*χ* ^2^ (df)	*χ* ^2^/df	*CFI*	*SRMR*	*RMSEA*	
1. Unconstrained Model: all loadings freely estimated across racial groups; direct path from exploration freely estimated			1.49	0.93	0.08	0.04
2. Mediation Constraint: path from ERI exploration to outcomes constrained to 0	1 vs. 2	19.18 (4)***	1.51	0.93	0.09	0.05
3. Fully constrained by racial background: All loadings constrained across racial groups	1 vs. 3	29.37 (9)***	1.51	0.92	0.10	0.05
**4. Partially constrained by racial background:** Freely estimated loading from ES to MA; all other loadings constrained across racial groups	**3 vs. 4**	**15.58 (8)**	**1.49**	**0.93**	**0.09**	**0.05**

*Note:* A null *χ* ^2^ difference test indicates that the more constrained model fits as well as the unconstrained model (model 1). The most parsimonious model was adopted at each step of testing, and the final model is bolded (model 4).

***p < 0.001.

### 5.4 Aim 2: Multicultural Attitudes and Civic Engagement

The model in which ERI exploration directly predicted multicultural attitudes and civic engagement was superior to the model in which this path was constrained to 0 (Model 1 vs. Model 4 in Table 3). Therefore, we computed indirect effects on outcomes via ERI exploration.

**FIGURE 2:**
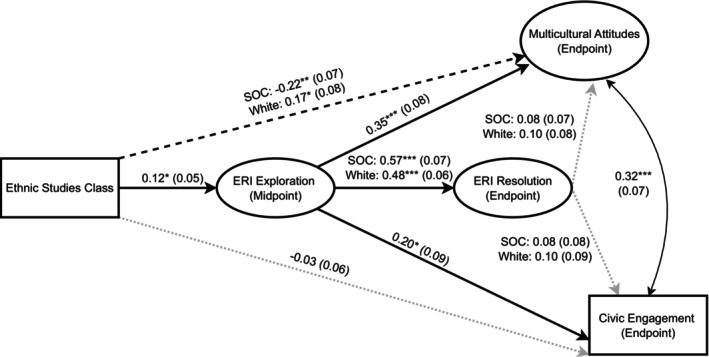
Structural equation model of relations between ethnic studies class and outcomes. Manifest variables appear as rectangles and latent variables as ovals. Standardized coefficients are reported for each path. Solid lines indicate significant paths that were equivalent across racial groups, including students of color (SOC) and White students. Dashed black lines indicate the path that differed between ethnic‐racial groups. Gray dotted lines indicate non‐significant paths. The gender covariate is included in the model, but not depicted. *p < 0.05, **p < 0.01, ***p < 0.001.

Consistent with H3, we observed positive indirect effects of the ES class on multicultural attitudes via ERI exploration for both students of color (indirect effect = 0.04, 95% CI [0.01, 0.10]) and White students (indirect effect = 0.04, 95% CI [0.01, 0.09]), which explained 21%–20% of the mediated effect respectively. However, multigroup SEM revealed that the direct effect between ES and multicultural attitudes differed significantly by racial background (Model 1 vs. 2 and 3; Table 3). The direct effect of ES was negative and significant for students of color, but positive and significant for White students (Figure 2; Table 4). This pattern indicated competitive mediation for students of color, wherein the direct and indirect effects have opposite signs (Zhao, Lynch Jr., and Chen 2010). In other words, ES course assignment predicted higher multicultural attitudes among students of color, only if it encouraged their ERI exploration, while it increased White students' multicultural attitudes, whether or not it promoted ERI exploration. Consistent with H4, the indirect effect of ERI exploration fully mediated the positive relation between ES class assignment and civic engagement for all students (indirect effect = 0.02, 95% CI [0.01, 0.06]).

**TABLE 4:** Estimates of paths in the final model for students of color and White students.

Path in the final model	Students of color		White students	
	*B* (SE)	*β* (SE)	*B* (SE)	*β* (SE)
Predictors of ERI exploration				
1. Ethnic studies class^a^	0.15 (0.06)*	0.12 (0.05)*	0.15 (0.06)*	0.12 (0.05)*
2. Female	0.37 (0.08)***	0.31 (0.06)***	0.21 (0.11)	0.16 (0.08)
3. Non‐binary	0.10 (0.19)	0.03 (0.06)	−0.19 (0.15)	−0.11 (0.08)
Predictors of ERI resolution				
4. ERI exploration	0.61 (0.09)***	0.57 (0.07)***	0.61 (0.09)***	0.48 (0.06)***
5. Female	−0.01 (0.09)	−0.01 (0.07)	−0.15 (0.16)	−0.09 (0.09)
6. Non‐binary	−0.67 (0.30)*	−0.19 (0.08)*	−0.21 (0.24)	−0.09 (0.10)
Predictors of multicultural attitudes				
7. Ethnic studies class	−0.35 (0.11)**	−0.22 (0.07)**	0.27 (0.15)*	0.17 (0.08)*
8. ERI exploration	0.43 (0.12)***	0.35 (0.08)***	0.43 (0.12)***	0.35 (0.08)***
9. ERI resolution^a^	0.09 (0.08)	0.08 (0.07)	0.09 (0.08)	0.10 (0.08)
10. Female	0.27 (0.11)*	0.19 (0.08)*	0.39 (0.16)*	−0.09 (0.09)
11. Non‐binary	0.45 (0.35)	0.11 (0.09)	0.96 (0.25)***	−0.09 (0.10)
Predictors of civic engagement				
12. Ethnic studies class^a^	−0.05 (0.11)	−0.03 (0.06)	−0.05 (0.11)	−0.03 (0.06)
13. ERI exploration^a^	0.29 (0.13)*	0.20 (0.09)*	0.29 (0.13)*	0.20 (0.09)*
14. ERI resolution^a^	0.11 (0.10)	0.08 (0.07)	0.11 (0.10)	0.10 (0.09)
15. Female	−0.18 (0.14)	−0.11 (0.08)	0.01 (0.19)	0.01 (0.10)
16. Non‐binary	−0.67 (0.43)	−0.14 (0.09)	−0.23 (0.28)	−0.09 (0.11)
Civic engagement ~ Multicultural attitudes^a^	0.16 (0.04)***	0.32 (0.06)***	0.16 (0.04)***	0.32 (0.07)***

Indirect effects	Estimate [95% CI]	Standardized estimate [95% CI]	Estimate [95% CI]	Standardized estimate [95% CI]
ES class → ERI exploration → ERI resolution	0.09 [0.02, 0.19]*	0.07 [0.01, 0.13]*	0.09 [0.02, 0.19]*	0.06 [0.01, 0.11]*
ES class → ERI exploration → Multicultural attitudes	0.07 [0.01, 0.15]*	0.05 [0.01, 0.09]*	0.07 [0.01, 0.15]*	0.05 [0.01, 0.09]*
Total indirect effect on multicultural attitudes	0.07 [0.01, 0.15]*	0.04 [0.01, 0.09]*	0.07 [0.01, 0.15]*	0.04 [0.01, 0.09]*
ES class → ERI exploration → Civic engagement	0.04 [0.01, 0.10]+	0.02 [0.01, 0.06]+	0.04 [0.01, 0.10]+	0.02 [0.01, 0.06]+
Total indirect effect on civic engagement	0.05 [0.01, 0.11]*	0.03 [0.01, 0.06]*	0.05 [0.01, 0.11]*	0.03 [0.01, 0.06]*

^a^ Indicates paths that were constrained to be equivalent across racial groups: students of color and White students.

^+^ *p* < 0.10.

* *p* < 0.05.

** *p* < 0.01.

*** *p* < 0.001.

### 5.5 Member Checking and Sensitivity Analyses

Findings were co‐interpreted with ES teachers and district partners to increase validity and impact of findings (Brush et al. 2020). In separate meetings with district leadership, ES teachers and students in an antiracism club, we found that our results and interpretations resonated with the districts' guiding framework and theory of change and teachers' observations. An alternative model suggested by an anonymous reviewer tested a latent construct involving both exploration and resolution as the mediator, given that high levels of both processes show the strongest links with positive adjustment (Umaña‐Taylor and Rivas‐Drake 2021). Direct and indirect effects were consistent with our primary model (Supporting Information S5).

## 6 Discussion

Amidst the fierce nationwide debate surrounding curriculum about racism and identity, this study used a natural experiment design to evaluate a new ES graduation requirement for high school students. Compared to students in a control class, a diverse group of 9th graders who took an ES class engaged in significantly more exploration of their ERI, resulting in stronger resolution of feelings about their ERI by the end of the semester (consistent with H1). Effects on ERI development were comparable for students of color and White students (contrary to H2). Furthermore, increased ERI exploration during the course explained more favorable multicultural attitudes (consistent with H3) and more frequent participation in their communities through civic engagement activities (consistent with H4). In the pilot year of this new curriculum and amidst the COVID‐19 pandemic, teachers effectively designed and delivered courses based on the district's general ES framework, which included explicit exploration of identity, structural analysis of racism, counternarratives of communities of color, and interdisciplinary learning that leads to action. Benefits for both students of color and White students suggests positive effects of district or state policies to expand access to ES courses, and the successful implementation points to the feasibility and scalability of such policies. Results also directly address concerns that these courses inflame racial animosity by showing just the opposite: students who explored their own ethnic‐racial background in the course saw diversity as an asset to society and reported eagerness to positively engage with people from other ethnic‐racial groups.

### 6.1 Strengthening the Evidence for ES

Since college students first advocated for ES classes in the 1960s, important research has linked these curricula to ERI development and student adaptation using primarily pre‐post or case study designs focusing on single ethnic‐racial groups (see review by Sleeter and Zavala 2020). Our study filled important gaps in the research on ES by using a natural experiment design to analyze a curriculum implemented by existing teachers in classrooms with students of diverse backgrounds. Natural experiments, which capitalize on quasi‐experimental randomization occurring in real‐world settings, can provide more robust evidence of causality and greater external validity than lab‐based or observational studies alone (Leatherdale 2019). This addresses the problem of “voltage drop” discussed in implementation science, in which promising results from tightly controlled, laboratory‐based efficacy studies show reduced effects when scaled up (Al‐Ubaydli, List, and Suskind 2019). While full randomization was not possible, it is notable that the effect size of the ES course on ERI exploration after 10 weeks of the semester was identical to that found in an RCT of the 8‐week Identity Project curriculum (*β* = 0.12 in the structural equation model from both studies) and the subsequent effect on resolution was slightly higher (*β* = 0.48 vs. 0.32 in the Identity Project; Umaña‐Taylor et al. 2018). As policymakers nationwide move to expand or ban ES, natural experiments offer a feasible way to evaluate policy impacts with minimal interference in school operations. These designs are especially suited to the current period of rapid innovation, but also backlash, around ES.

### 6.2 Developing ERI and Global Competence in Schools

ERI was first conceptualized as a protective factor among communities of color for resisting racism, and decades of research bore out these predictions (Umaña‐Taylor and Rivas‐Drake 2021). This study contributes to a growing body of evidence that ERI can also be understood and studied as a universal construct linked with adolescent adaptation and global competence in diverse societies (Jang, Schwarzenthal, and Juang 2023; Umaña‐Taylor 2023). Results showed that the ES class increased both ERI exploration and resolution for students of color and White students. Additionally, mediation analyses identified increased ERI exploration as a key mechanism of increasing civic engagement and multicultural attitudes following the ES course.

As youth develop in multicultural societies grappling with deeply entrenched racism, exploring their ERI supports their motivation to engage with diverse others and to feel a sense of agency to shape society (Rivas‐Drake et al. 2022). In diverse ES classrooms and communities, the active processes involved in exploration (e.g., seeking information, engaging with one's identity) may simultaneously foster interactions and identification with outgroup members facing similar forms of oppression and heightened awareness of news or civic engagement opportunities (Fish et al. 2021). Our results about the centrality of exploration for global competence are consistent with findings among a diverse group of college students that ERI exploration, but not resolution, was associated with the development of an “ally identity,” involving identification with the struggles of other groups and engagement in sociopolitical behaviors (Fish et al. 2021). A commitment to lifelong exploration is also a key component of developing a healthy antiracist identity in Helms' (2019) model of White racial identity development, since White students become aware of the ways that they are personally involved in challenging or maintaining racism and White supremacy. Importantly, the centrality of exploration in our findings also supports the district's guiding framework for ES, as explicit exploration of ERI and structural analysis of racism fostered higher rates of action by the end of the semester.

Interestingly, while White students experienced both direct and indirect increases in favorable multicultural attitudes, improved attitudes among students of color were fully mediated by their increased ERI exploration (competitive mediation; Zhao, Lynch Jr., and Chen 2010). This suggests that personal ERI development is crucial for supporting the multicultural attitudes of students of color, who experience substantial identity‐related threats in schools and may have valid, self‐protective reasons for withdrawing from other groups. This also parallels findings in the parental ethnic‐racial socialization literature that different strategies are associated with either risk or protective effects (Hughes et al. 2006; Umaña‐Taylor and Hill 2020). For example, the ES frameworks' guidelines around identity exploration and positive counternarratives are akin to the strategy of *cultural socialization*, which is robustly linked to protective and promotive effects. The critical analysis of racism guideline could have functioned as *preparation for bias* or *promotion of mistrust*, which may enhance risk for poor mental health or withdrawal from outgroup interactions and peers in the absence of other forms of socialization (see review by Umaña‐Taylor and Hill 2020). Supporting ERI development in parallel with analyzing structural inequities seems crucial for maximizing the benefits of ES for multicultural attitudes and civic engagement, especially for students of color. Indeed, this interpretation resonated with district leadership and ES teachers during member checking, and at their request, study staff produced a toolkit to support teachers' integration of ERI development into future iterations of the course.

White parents, who may otherwise avoid explicit discussions of race with their children, often select schools aligned with their racial socialization goals (i.e., exposure to diversity) or that mirror their biases (i.e., preference for majority White schools; Hagerman 2020). In the current study, White students were demographic minorities at all four included schools, creating situations of high intergroup contact. ES classes that explicitly discuss the construction of race and current effects of racism challenge norms of Whiteness, including avoidance of racial discomfort and colorblind racial ideology (Neville et al. 2013; Roberts and Rizzo 2021). This type of ethnic‐racial socialization from teachers could be especially important for creating a positive school racial climate in a racially diverse school (Wang, Henry, and Del Toro 2023). Supporting teachers whose classes are comprised of students with vastly different lived experiences and family ethnic‐racial socialization practices is a critical future direction for widespread implementation of ES lessons in diverse classrooms.

The impacts of ES courses within schools could expand beyond individual students and into the broader school and community through students' multicultural attitudes and civic engagement. In fact, this was an explicit goal of the ES framework, which focused on interdisciplinary learning about racism and social movements that would lead to action. First, positive school climate could arise from school‐based racial socialization which encourages students to understand their own ERI and adopt a multicultural approach to diversity (Wang, Henry, and Del Toro 2023). These attitudes and the school climate they engender at the student‐body level could reduce rates of discrimination and harm to students of color, which peak during the early years of high school (Alinor, Simons, and Leigh 2023; Wang and Yip 2020). Second, increased civic engagement following these courses prepares students to carry on a long history of youth activism, which includes recent examples of youth leadership in social movements for climate justice, gun safety, and immigration reform (Diemer et al. 2021). As a direct case in point, Cabrera et al. (2013) documented student‐led teach‐ins to protest the ban of a successful Mexican American Studies program in Tucson, AZ. In summary, our results suggest that the individual benefits of ERI identified in previous work are likely precursors to cascading effects on the school climate and communities in which adolescents live. Future research should explicitly explore the effect of ES courses on broader levels of adolescents' social ecologies.

### 6.3 School and Policy Implications

The UCLA School of Law has documented efforts to ban school‐based discussion of critical race theory and related topics in all but one of the 50 U.S. states, yet other districts and states are actively expanding or requiring new ES courses that center discussions of race and racism (Alexander 2023). In harmony with existing research (Sleeter and Zavala 2020), our results reinforce the benefits of ES curriculum and other programs that encourage ERI reflection in schools. Far from increasing racial animosity or lowering patriotism, results suggest that students with a better understanding of their ERI are more likely to embrace diversity and to be active participants in their communities. Ongoing policy evaluation utilizing natural experiments in schools can meet this political moment by providing direct evidence to families and school boards about the potential benefits and absence of harm for their students.

Additionally, our results speak to the feasibility of implementing ES classes using a broad framework and engaging existing teachers of different backgrounds to implement the courses. All ES and control teachers at Schools 1 and 2 were White and taught a broad ES class that included the histories of many different ethnic‐racial groups, representing a majority of the sample, while teachers at Schools 3 and 4 were more likely to be teachers of color and to teach courses that were specific to their own heritage or the heritage of students at that school (e.g., School 4 where the majority of students shared Somali American heritage). The broad framework, relative to a fixed set of readings or topics, allowed teachers to tailor course content to their students' interests and may provide a generalizable model for other districts.

Schools that adopt these curricula have a chance to harness students' increased motivation for civic engagement through facilitating opportunities and celebrating student voice. At the same time, evidence shows that school policies and procedures often subvert or even punish students for activism. For example, student walkouts are often viewed and punished as truancy, rather than as legitimate expressions of students' first amendment right to free speech (Brown 2012). Youth have been a consistent part of social movements and change for generations, and school policies that foster, rather than suppress, their participation can support adolescents in developing effective methods for lifelong civic engagement (Torney‐Purta 2002).

### 6.4 Limitations and Future Directions

The current study has notable strengths, including the testing of pre‐registered hypotheses using a natural experiment design. Nevertheless, results should be interpreted in light of several limitations. First, this study was unable to fully randomize students to their course condition, and baseline data were unavailable to substantiate similarity between the groups on ERI or other outcomes prior to the course. While quasi‐randomization was used to address this limitation, bias is reduced in natural experiments that include baseline data (Wong, Steiner, and Anglin 2018), which should be a priority of replication efforts. Additionally, while we found no evidence of clustering within classrooms, replications in larger samples allowing for multilevel modeling could produce more robust estimates of each effect.

Second, there were high levels of missing data in the current sample. Data were collected amidst a turbulent year with staffing changes and COVID‐19‐related impacts on teachers and students, which limited survey retention. While data were MCAR across the midpoint and endpoint and midpoint levels of all variables were not associated with missingness, students at one school that experienced staffing changes were more likely to be missing at follow‐up. It is possible that the added stressors on teachers at this school could have impacted both survey retention and course effects; in this scenario, missing surveys from these students may overestimate the effects observed in our study. To maximize power and take advantage of all available data, we followed recommendations to use FIML and include auxiliary variables that predict missingness (Dong and Peng 2013; Graham 2003).

Third, our analyses clustered students from a broad range of ethnic‐racial backgrounds into one group (i.e., students of color). This modeling choice allowed us to broadly understand differences in how students of color and White students experienced ES, but it obscured potentially meaningful intragroup differences. The impact of discussing race may also differ by the ethnic‐racial background of the teacher, which was not investigated here. Additionally, the included schools shared some characteristics, including an urban, Midwestern setting and student bodies comprised of a majority of students of color, which may limit the generalizability of our findings to other similar settings. Replication in other areas, such as suburban or rural schools with different demographics, should be a priority as ES expands at a state level (e.g., California State Board of Education's Ethnic Studies Model Curriculum).

Finally, the curriculum was guided by a district‐determined framework, but not a specified curriculum. Teachers' fidelity to the ES framework was not assessed, limiting our ability to compare these findings to other ES evaluations or to identify shared, active ingredients. Future examinations of teacher‐ or classroom‐level variability would be possible with a larger sample of classrooms and could yield insight into critical mechanisms of the course's impact.

## 7 Conclusion

ES courses encourage ERI development and critical examination of history and social movements, preparing students to engage in today's diverse democratic societies. Using a natural experiment design to evaluate a new ES high school graduation requirement, this study found that students who develop a deeper understanding of their own ERI through taking an ES course embrace multiculturalism and participate in their community through civic engagement activities. Results directly refute fears that talking about race and racism stokes animosity by showing that exploring one's own identity improves students' eagerness to learn from and interact with people from different backgrounds. Indeed, ES courses are one way of addressing the root psychological causes of U.S. racism and encouraging participation in civic, social, and political movements for racial justice (Roberts and Rizzo, 2021; Leslie et al. 2020; Rivas‐Drake et al. 2022). Ultimately, students taking ES classes are well‐prepared to thrive in a diverse society and to shape a more equitable future through their attitudes and actions.

## Supporting information


Data S1.

## Data Availability

The data necessary to reproduce the analyses presented here are not publicly accessible. Data come from the administrative records of a school district including children under the age of 18. Per our data use agreement with the school district, only researchers who have been approved by the districts' internal research review process may access data. Researchers may seek independent approval to access survey and administrative data used in this study. Code for analyses is available from the first author upon request. The materials necessary to attempt to replicate the findings presented here are publicly accessible. Materials and the pre‐registration for the current study are available at the following URL: https://osf.io/bdkty/?view_only=7174dde1668842e4a6e575f56091c0c0.
