# Supplementary material for: Promoting Identity Development, Multicultural Attitudes, and Civic Engagement Through Ethnic Studies: Evidence From a Natural Experiment
Source: Child Dev. 2025 Jan 20;96(3):966–79. doi: 10.1111/cdev.14219 (PMC12023817; doi:10.1111/cdev.14219)
Supplement: Supplementary file 1 — Data S1. [file CDEV-96-966-s001.docx]

Supplementary material for Gillespie, S. Morency, M. M., Fajemirokun, E., & Ferguson, G. M. (2025). Promoting Identity Development, Multicultural Attitudes, and Civic Engagement Through Ethnic Studies: Evidence From a Natural Experiment. *Child Development.*

**Supplement 1 (S1). Factor Structure and Measurement Invariance**

**Ethnic Racial Identity**

Confirmatory factor analysis (CFA) supported a single factor structure for the ERI exploration subscale at both the midpoint and endpoint surveys; however, the lone reverse-scored item had very low loading onto the latent factor (β <0.30) among both students of color and White students. Removing this reverse-scored item increased values of Cronbach’s alpha, so we proceeded with a 6-item subscale after confirming acceptable fit (χ^2^/*df* = 1.54; *RMSEA* = 0.05; *CFI* = 0.99). We found configural invariance across students of color/White students at both midpoint and endpoint (*p >* 0.05 for Δχ^2^). Cronbach’s alphas were acceptable at midpoint (White α = 0.89; students of color α = 0.86) and endpoint (αs = 0.87 & 0.82, respectively).

A CFA also supported a single factor structure at both timepoints for the 4-item ERI resolution subscale and all items loaded strongly onto the latent factor (β > 0.40). Scalar invariance was attained (Δχ^2^(6) = 11.46, *p* = 0.08). Cronbach’s alphas were high at midpoint (White α = 0.89; students of color α = 0.90) and endpoint (α = 0.92 & 0.90, respectively).

**Multicultural Attitudes**

CFA of the Multicultural Attitudes items confirmed that all items load strongly onto a single latent variable (β > 0.40). Measurement invariance testing established metric invariance based on racial background (Δχ^2^(12) = 16.50, *p* = 0.17).

**Supplement S2. Comparison of Ethnic Studies and Control Group after Quasi-Randomization**

While the omnibus χ2 test for racial background across ES and control groups was not significant (11.24 (6); *p* = 0.08), given the marginal effect, further examination of standardized residuals for values exceeding the critical threshold of 1.96 indicated that Black students were slightly more likely to be enrolled in the ES class than the control (standardized residual = -2.1), while all other groups were approximately equal (standardized residuals <1.1). Black students were overrepresented at School 1, where all students took ES, therefore this is attributable to the overarching school policy around universal ES enrollment rather than a selection effect of opting into the class.

Quasi-randomization was also checked within Schools 2-4 to establish whether each school’s procedure for assigning ES or control classes produced comparable groups. Binomial logistic regression indicated no significant effect of any coefficients in the omnibus test (*p* values > 0.45) when race, receiving English Language Learner services, gender, and middle school GPA and attendance were entered as predictors of class enrollment. Finally, χ2 tests were used to examine students’ other course enrollment, given that academic tracking based on other subjects could have influenced course enrollment; results of these tests were nonsignificant at the three schools with variation in enrollment (*p* values 0.13-0.39 across tests). At all schools, the majority of students took Intermediate Algebra (ranging from 59.6% to 94.7%), 9^th^ grade English (45.3% to 92.1%), and Physical Science (94.7% to 97.4%). Taken together, results support the inference that class assignment (ES vs. control) was determined independent of students’ race, middle school academic performance, academic track, English proficiency, and gender.

**Supplement S3. Author Positionality**

The first author is a U.S.-born White woman, the second author is a U.S.-born Haitian American woman raised in the Midwest, the third author is a U.S.-born Nigerian American woman raised near the site of the study, and the anchor author is a Jamaican American woman born in Jamaica. Each author experienced unique influences on their own ethnic-racial identity as a result of their positionality, region of origin, family socialization, and school experiences, which informed hypotheses about the effects of ERI development for students of color and White students. Authors attended a mix of private and public K-12 schools in their country of origin. None had ES courses offered during their K-12 education, but many opted into these courses in college, shaping favorable expectations about the potential benefits of ES classes.

**Supplement 4 (S4). Data Available by Wave, Class Type, and Racial Background**

|  |  | Midpoint Data Present | | Endpoint Data Present | |
| --- | --- | --- | --- | --- | --- |
|  |  | *n* | *%* | *n* | *%* |
| Ethnic Studies | Students of Color (*N* = 243) | 193 | 79.4% | 134 | 55.1% |
|  | White students  (*N* = 109) | 100 | 91.8% | 48 | 44.0% |
| Control Group | Students of Color (*N* = 112) | 88 | 78.6% | 36 | 32.1% |
|  | White students  (*N* = 69) | 61 | 88.4% | 20 | 29.0% |

*Note*. Students who provided any data at the midpoint or the endpoint were included in analyses.

**Supplement 5 (S5). Sensitivity Analysis of Mediation by Composite Ethnic-Racial Identity**

Our sensitivity model tested a composite of exploration and resolution at the midpoint and endpoint as mediators of multicultural attitudes and civic engagement. The model fit was inferior to our primary model (*CFI* = 0.87, χ^2^/*df* = 6.32, *RMSEA* = 0.05, *SRMR* = 0.09), likely attributable to our earlier CFA that confirmed exploration and resolution operate as separate factors in our data. The composite variable did mediate higher multicultural attitudes (indirect effect = 0.03, 95% *CI* [0.01, 0.07]) and civic engagement (indirect effect = 0.03, 95% *CI* [0.01, 0.07]).

**
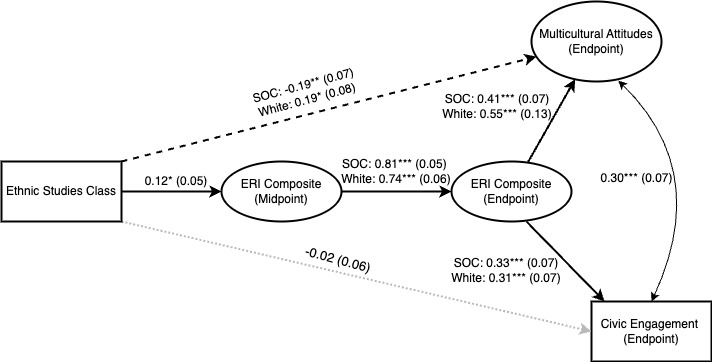
**

*Note.* Manifest variables appear as rectangles and latent variables as ovals. Standardized coefficients are reported for each path, with standard errors reported in parentheses. Solid lines indicate significant paths that were equivalent across racial groups, including students of color (SOC) and White students. Dashed black lines indicate the path that differed between ethnic-racial groups. Gray dotted lines indicate non-significant paths. The gender covariate is included in the model, but not depicted.

* *p* < 0.05, ** *p* < 0.01, *** *p* < 0.001
